# Alcohol as a Cause of Disease

**Published:** 1907-09

**Authors:** N. P. Jelks


### ALCOHHOL AS A CAUSE OF DISEASE.

By N. P. Jelks.

It is not my purpose in this paper, to discuss the use of Alcohol either as a food or medicine in the treatment of disease, nor to go into the details of its devilish effects upon the moral nature of the confirmed inebriate. I will only allude to the thousands of murders, the tens of thousands of thefts, and robberies committed annually which are truthfully laid to its door. To the brutal violence committed by the drunkard upon the faithful heart broken wife whom he promised at the altar to love, honor and support. To the neglect and cruelty shown his children. To the innumerable hosts of our young men, the best blood of the land who are led away from the path of virtue and rectitude by this fell destroyer into the gambling hell and house of prostitution. Verily Whiskey and Women go together and what is the result. Syphilis and gonnorrhoea have in many cases wrecked the health of these victims forever. They have become drunkards and vagabonds a disgrace to their families, a pest to society. I will endeavor as briefly and concisely as possible to bring to your remembrance some of the most important diseases to which the constant drinker is subject; I will note:

1st: Its effect on the stomach;

2nd; Its effect on the liver;

3d: Its effect on the heart and blood vessels;
4th: Its effect on the lungs;
5th: Its effect upon the brain and nervous system generally;
6th: Its effect upon the kidneys;
7th: Its effect upon the muscles;
8th: General effect.

After continued daily use of alcohol in one or more of its combinations, the victim suffers from catarrh of the stomach. The glands become atrophied, causing a most distressing form of dyspepsia often followed by inability to retain anything taken into the stomach early in the morning. The drunkards vomiting has come. Many times the poor fellow takes his morning dram, only to eject it again, and again, until a drink will stick, when he is all right once more, and goes on his downward course until next morning when there is a repetition of the same terrible suffering.

There was once a poor drunkard in my town, who was actually compelled to hold his nose while swallowing the whiskey. The smell of the stuff was more than his stomach would bear.

The second evil noticed is the effect of alcohol upon the liver of the constant drinker. At first conjestion of the organ takes place causing enlargment. This finally terminates in atrophy. Cirrhosis, that incurable disease, rightly denominated the "Gin drinker's liver." has seized its victim.

This is soon followed by ascites, and in a few short months death steps in to claim its own.

The third step in the tragedy, may be mentioned the effect upon the heart and blood vessel.

The heart is enlarged from fatty degeneration. The endocardium and coats of the arteries are sometimes filled with altheromatous deposits. The capillaries are conjested and the veins become varicose.

Sudden death from Heart Failure is not uncommon.

The fourth thing noticed is the effect of alcohol upon the lungs of the steady drinker. Chronic bronchitis is often found.

Also emphysema, hypostatism, pneumonia, and last but not least phthisis, that scourge of mankind is oftentimes lighted up, to help break the brittle thread of Life.

The fifth step, is the effect upon the brain and nervous system generally. The brain wastes, shrinks, and the nerve cells waste away, as a consequence the mind is weakened, both the intellect and the moral nature are dulled. The man has again become a child, and if delirium tremens with all of its horrible and hellish accompaniments is not sufficient to overwhelm the unfortunate fellow, permanent insanity steps in to help him on to a premature death. If neither of these are sufficient to do the work, neuritis with its terrible suffering, followed by paralysis adds to the horror and danger of the situation.

The sixth thing noticed, is its effect upon the kidneys.

Bright's disease in the cirrhotic form has taken place, and if the foregoing list of diseases it not sufficient to hasten the inevitable end rapidly enough, uraemic convulsions occur, and soon all is over.

The seventh pathological change is the condition of the muscles. They are soft, flabby, and weak. The man walks with a decided effort. The poor fellow is on his last legs. He is too weak to work, to feeble to walk; his mind is almost gone, and as we look upon this wreck of fallen humanity, with his red eyes, and bloated face, and simple countenance, we exclaim "Poor Fellow," "Whiskey did it" "Whiskey Did It All, Yes." Whiskey has ruined him physically, financially, and morally. His health and strength are gone, when he could have been strong and vigorous. He is a pauper when he could have been above want. His sense of right and wrong is so blunted, that if he were not so weak, he would delight once more to beat his faithful wife, and mistreat his ragged children. He has lived a drunkards life, has brought those, whom he should have loved, honored and supported, down to poverty and disgrace.

His wife is cowed, and his children have grown up in ignorance and poverty. And now all that is left for him to do, is to fill a drunkards grave, and a drunkards hell. "No drunkard shall enter the Kingdom of Heaven."

How strange that sensible men, especially those, who know all about the effects of this mighty instrument for evil, will indulge their appetite. For at last it "stingeth like an adder and biteth like a serpent."

